# Innovative Strategies for Public Health Training in the Asia Pacific: Insights From Experience and Evidence

**DOI:** 10.1177/10105395241301817

**Published:** 2024-11-29

**Authors:** Philip R. A. Baker, Julie-Anne Carroll, Daniel Demant

**Affiliations:** 1 School of Public Health and Social Work, Faculty of Health, Queensland University of Technology, Brisbane, QLD, Australia; 2 Australian Centre for Health Law Research, Queensland University of Technology, Brisbane, QLD, Australia; 3 Faculty of Medicine, Universiti Teknologi MARA, Selangor, Malaysia; 4 Graduate School of Public Health, Yonsei University, Seoul, South Korea; 5 School of Public Health, Faculty of Health, University of Technology Sydney, Ultimo, NSW, Australia

**Keywords:** public health education, innovation in teaching, blended learning, peer-assisted learning (PAL), digital learning technologies

## Abstract

The past decade has seen a rapidly changing landscape in priority areas for public health globally and, as such, across the teaching and learning curriculum for tertiary education in health sciences. The nature of some of these changes has led to pedagogical challenges in higher education that require transformative, interactive, and virtual modes of delivery and knowledge facilitation not previously seen. The COVID-19 pandemic, climate change, increasing health disparities, and a shift to a focus on noncommunicable diseases has merged with the changing nature of social, cultural, and technological preferences of the generations living through such times to see an increasing need in more viable teaching solutions for these “wicked problems.” This article outlined key innovations empirically demonstrated to meet these challenges through nuanced responses to increasingly disrupted approaches to linear delivery of content and a shift toward bite-sized, interactive, reflexive modes of achieving learning objectives.

## What We Already Know

- Public health training often relies on traditional didactic methods that may not effectively address complex, real-world challenges.
- The rapid shift to online learning during the COVID-19 pandemic highlighted the need for more flexible and innovative teaching methods.
- Active learning and digital technologies, such as peer-assisted learning and audience response systems, have shown promise in enhancing student engagement and learning outcomes.

## What This Article Adds

- Proposes blended learning approaches that integrate digital tools, such as Padlet and AI-guided learning, to improve public health education.
- Demonstrates how innovative strategies like mock randomized controlled trials can provide hands-on learning experiences in public health.
- Explores the impact of using collaborative digital workspaces to foster deeper learning and critical thinking among public health students.

## Introduction

The landscape of public health education in the Asia-Pacific region is rapidly evolving, driven by the need to address current and emerging challenges. These challenges include the rapidly aging population, the rising prevalence of noncommunicable diseases, and persistent and emerging infectious diseases, such as COVID-19, avian influenza and Mpox. The region’s public health challenges are complex and diverse, primarily due to its population, economic disparities, and varied health care infrastructures. More effective prevention and control programs are needed, particularly health sector responses. Public health practitioners, at the forefront of these efforts, apply skills in advocacy, planning, and evaluation. Furthermore, graduates must be confident in their knowledge and skills in the increasingly complex epidemiological mapping of diseases, geographic information systems, multilevel analysis, and digital health monitoring and interventions. Contemporary public health practice also requires skills in health promotion and are to be confident in the knowledge and skills they have in the protection to deliver quality, responsive services.^1^ As a result, there is a growing focus on developing public health training at both the graduate and postgraduate levels.

## Context and the Importance of Innovation in Public Health Education

Public health education in the Asia-Pacific must respond to challenges by keeping content relevant, incorporating contemporary pedagogy, and integrating suitable technologies. Earlier works have mapped out future trends and provided examples of practice and the eight domains of core competencies in public health.^1^ Public health training must move beyond passive classroom methods to engage and challenge students and academics through active, flexible learning, and realistic problem-solving.^1,2^ Accessing global training resources can enhance staff and student skills while complementing class-based teaching. The global pandemic of COVID-19 created a sudden disruption that immediately shifted teaching online, requiring both educators and students to innovate with e-learning and become knowledgeable about the rising trends in m-health apps and platforms. It has been suggested that innovation through e-learning and digitalisation was not merely a crisis response but a necessary evolution for the future of sustainable public health education.^3^ While massive open online courses (MOOCs) have the potential to deliver public health knowledge and skills training effectively and at low cost, a recent study showed an absence of offerings from institutions in low- and middle-income countries in important contemporary areas such as outbreak and pandemic management.^4^ In the Asia-Pacific, where educational challenges, including resources and infrastructure, are diverse, digital, and blended learning models could be more widely adopted to enhance public health education and professional development.

Although the move to online teaching during the pandemic facilitated continued learning, it did not necessarily reduce passive didactic approaches, as many traditional lecture formats persisted on platforms such as Teams and Zoom. However, some educators successfully adapted by integrating more interactive tools and techniques. The extent of the postpandemic return to passive on-campus lectures remains uncertain, with concerns of declining in-class attendance. In contrast to traditional didactic approaches, active learning means students engage in activities that develop skills and use higher-order thinking. Advances in technology paired with pedagogy can lead to new tools that enhance student learning experiences.^2^ Audience response technology (ART) has long engaged learners, starting with wireless “clickers” and now integrated into web and app technologies on personal devices.^2^ Polling technology is now integrated into teaching platforms like Teams, Zoom, and Padlet.

To address the region’s emerging challenges, there is an opportunity to implement modern pedagogy with evolving technology within a blended learning approach that promotes interactive environments. Examples of innovative teaching and learning strategies with learning skills by pedagogy can be found in Table 1. In Australia, online collaborative spaces such as Wikis and Padlet have been used for reflective learning in public health topics, such as women’s health and gender equity and human health.^5^ While Wikispaces ceased operations, Padlet achieves similar teaching and learning objectives using iterative, collaborative, and competitive peer-to-peer learning through observation and modeling. This has been analyzed and evaluated using Bandura’s social learning theory (SLT), self-efficacy, and social constructivist theory.^5^ For example, social constructivist theory emphasizes learning as a social process where knowledge is constructed through interactions and collaborations. Padlet allows such processes to manifest in a way that aids deep and sustained learning over complex social issues.^6^ This approach fosters deeper understanding in public health education by encouraging students to discuss, debate, and build on each other’s ideas.^5^ This aligns with the region’s health problems, often requiring collaborative problem-solving.

**Table 1.** Pedagogy and Innovative Learning Strategies.

Active learning pedagogy Involves students actively engaging in their learning through discussion, problem-solving, or hands-on activities rather than passively receiving information. *Social constructivism and constructivist learning*	
Strategy	Description
Peer-learning	Students learning from each other through discussions, feedback, and collaborative problem-solving. This approach encourages active participation and deepens understanding by allowing students to explain concepts to their peers in supportive environments. Cultivates collaborative and interpersonal skills.
Flipped classrooms	Students engage with lecture content outside of class, often through videos or readings, allowing class time to be dedicated to interactive activities. This fosters active learning by shifting the focus from traditional lectures to collaborative and problem-solving tasks in face-to-face sessions.
Experimental learning pedagogy Learning takes place through experience, reflection, and application of knowledge in real-world situations. *Experiential Learning Theory (ELT)*	
Experiential learning (e.g., Mock-RCTs)	Focuses on hands-on, real-world experiences students engage in activities such as simulations, internships, or fieldwork. Learning by doing, allowing students to apply theoretical knowledge in practical settings. Develops data literacy and epidemiological skills.
Technology-enhanced learning pedagogy Technology is used to enhance learning through engagement, motivation, and interactivity. *Motivational Learning, Formative Assessment*	
Game-based learning (e.g., gamification, virtual reality)	Integrates elements of games or virtual simulations to make learning more engaging and interactive. Uses challenges, rewards, and real-life scenarios to motivate students and enhance their understanding of complex concepts. Fosters motivation, leadership, and decision-making through scenarios that mimic public health challenges, helping students build resilience and strategic thinking.
Polling (e.g., Kahoot, Mentimeter)	Engages students through live quizzes, surveys, or feedback sessions during lessons. These platforms allow educators to assess student understanding in real-time and encourage active participation in both face-to-face and online environments. Build evaluation and assessment skills by enabling students to respond, practice formative assessment, and track progress.
Collaborative learning pedagogy Students learn by working together in teams, developing both subject matter knowledge and interpersonal skills. *Problem-based learning (PBL), Cooperative learning*	
Collaborative digital workspaces (e.g., Padlet)	Provide an interactive platform for students and educators to share content, ideas, and feedback in real-time. These tools facilitate teamwork, allowing participants to contribute to projects and discussions from different locations. Enhance communication and teamwork skills, crucial for managing public health initiatives requiring coordination across diverse stakeholders.
Personalized and adaptive learning pedagogy Tailors instructions to meet individual learners’ needs by adjusting content, pace, and learning strategies. *Adaptive learning approaches*	
Artificial intelligence (AI)-guided and -enhanced learning	Uses AI to personalize educational experiences, adapting content to meet individual student needs and progress. This approach also includes AI-enhanced tools, such as chatbots and virtual tutors, to support learning outside of traditional classroom settings. Develops adaptive problem-solving abilities.

The table illustrates pedagogy and innovative approaches, typically assessed by cross-sectional surveys, and retrospective evaluation.

Emerging challenges, such as threats to assessment integrity, including contract cheating also drive the need for innovation and authenticity in public health learning and teaching. The prevalence of contract cheating, outsourcing, and essay mills has increased in recent years. Factors like student circumstances, perceived difficulty, and the growth of online services create an environment where students may rationalize cheating.^7^ More recently, artificial intelligence (AI) chatbots, such as ChatGPT, have presented a new form of contract cheating. A survey of 337 Australian university students found that over a third had used chatbots for assessment assistance.^8^ This study provides insights via the findings that spanned the complex psychosocial underpinnings and contexts in which Australian students accept and reject AI. Interestingly, participants did not view this as a breach of integrity, and the reasoning seems similar to the reasons reported for accessing essay writers. Given the widespread use of such tools, educational institutions must reform policies. While chatbots pose challenges, they also offer opportunities to rethink assessment design, creating more authentic, real-world assessments that foster deeper learning.^8^ This highlights the need for ongoing consultation and observation of students’ evolving learning behaviors in the postpandemic context amid growing reliance on social and learning technologies that shape new, shared cultures in previously uncharted in higher-learning.

## Challenges and Barriers to Innovation

Challenges and barriers to innovation in learning and teaching must be overcome for public health education to evolve and remain relevant. Institutional resistance often arises from deeply ingrained traditions, rigid structures, and reluctance to adopt new practices or technologies. This resistance obstructs innovation by limiting support for new methods, providing inadequate training, and focusing on preserving the status quo. When institutions resist change, the adoption of innovative teaching approaches is slowed, reducing the ability to meet evolving educational needs and stifling creativity.^9^ A systematic review of the determinants of innovation behavior in public health higher education, identified that a lack of institutional support and a culture that discourages risk-taking prevent educators from engaging in innovative practices.^10^ Teachers are less likely to adopt new methods if they feel their institution does not value or reward innovation.

Inconsistent professional development leaves educators unprepared and reluctant to try new techniques.^11^ Other work environment factors, such as bullying, create a hostile atmosphere that stifles creativity and innovation.^12^ Levy^13^ highlights that academic bullying harms individual well-being and impedes academic progress, reducing educators’ willingness to innovate.

With a supportive environment for creativity, the possibilities for addressing these challenges are vast. Immersive technologies, such as virtual reality (VR) and augmented reality (AR), could be used to simulate public health scenarios such as outbreak management, although the high costs may be a barrier. Initiatives such as the Asia-Pacific Consortium for Public Health’s annual conference and other regional activities provided through similar organizations offer valuable forums for the region’s academics to share practical solutions for improving learning and teaching practices.

Successful innovative strategies combine sound pedagogy with creative approaches to address challenges. An example is peer-assisted learning (PAL) in epidemiology, where students in postgraduate epidemiology use peer instruction to review each other’s work critically.^14^ This method encouraged collaboration, mutual feedback, and active engagement with material, fostering both personal and collective growth. Peer-assisted learning has also been found to reduce the risk of contract cheating like essay writers and chatbots.

Another innovative example is the mock randomized controlled trial (RCT), where participants engaged in a simulated trial using candies as mock therapy and ART for data collection.^15^ This hands-on, interactive approach enables students to apply epidemiological concepts in real time, enhancing their understanding and critical appraisal skills. The student perspective is essential to ensuring equality, as the ease of use of technology, its accessibility, and its acceptance may create learning barriers.

## Conclusion

Developing and adopting innovative strategies in public health education, particularly within the Asia-Pacific region, are essential to address emerging challenges such as an aging population, noncommunicable diseases, and emerging health challenges. The combined application of sound pedagogy, such as PAL and digital technology, can create new approaches, such as the RCT simulation. There is an opportunity for public health academics to be innovative to improve learning outcomes, enhance key skills, and foster deeper engagement. Future research should explore how institutions can create a culture and opportunities that support innovative practice. Finally, continuous evaluation is essential for both new and existing strategies to ensure they effectively meet learning objectives.
